# The value of international volunteers experience to the NHS

**DOI:** 10.1186/s12992-019-0473-y

**Published:** 2019-04-23

**Authors:** B. Zamora, M. Gurupira, M. Rodes Sanchez, Y. Feng, K. Hernandez-Villafuerte, J. Brown, K. Shah

**Affiliations:** 1 Office of Health Economics, Southside 7th floor, 105 Victoria Street, London, SW1E 6QT UK; 2 Health Education England, University of Leeds, Willow Terrace Road, LS2 9JT Leeds, England; 3 Present Address: Centre for Primary Care and Public Health, Queen Mary University of London, London, UK; 4 Present Address: German Cancer Research Center (DKFZ), Heidelberg, Germany

**Keywords:** Global engagement, International volunteers, Health partnerships, Productivity

## Abstract

**Background:**

Global Engagement works with health partnerships to establish workforce and educational translation on a global scale to support the National Health Service (NHS). There is growing evidence on how international experiences (through volunteering, exchanges and placements) benefit the NHS through an innovative workforce that develops international best practice and promotes lifelong learning. Most of this evidence has been captured though surveys to returned international volunteers. However, there is limited evidence about how to quantify the value that returned international healthcare volunteers bring back to their country of residence.

**Methods:**

This paper identifies the various benefits to the NHS from returned international healthcare volunteers. The outcomes from returned international volunteers, which have been identified as relevant form a NHS perspective, are linked to three key areas in a multisector analytical framework used by the World Bank to evaluate labour market programmes: (1) Investment climate and Infrastructure, (2) Labor market regulations and institutions, and (3) Education and skills development. The monetary value of these outcomes is quantified through productivity indices which capture the economic value that the achievement of these outcomes have on the quality of the NHS labor force. This model is applied to a dataset of international volunteers provided by the Global Engagement health partnerships.

**Results:**

The results suggest that international volunteering generates average productivity gains of up to 37% for doctors and up to 62% for nurses. Average productivity gains estimated from health partnerships data vary depending on duration of volunteering periods and occupational category mix.

**Conclusions:**

Our analysis offers a value for money rationale for international volunteering programmes purely from a domestic and NHS perspective. The valuation method considers only one of the aims of international volunteering programmes: the development of the existing and future NHS workforce. Broader benefits for health system strengthening at a global level are acknowledged but not accounted for. Overall, we conclude that if the acquisition of volunteering outcomes is realised, the NHS can accrue a productivity increase of between 24 and 41% per volunteer, with a value ranging from £13,215 to £25,934 per volunteer.

**Electronic supplementary material:**

The online version of this article (10.1186/s12992-019-0473-y) contains supplementary material, which is available to authorized users.

## Background

The Global Engagement (GE) is a Health Education England (HEE) programme working to establish workforce and educational translation on a global scale to support the National Health Service (NHS). This is being done through programmes focused on international recruitment, global placements, evaluation and research, international volunteering and health partnerships.

GE is committed to evaluating the benefits of these programmes as a part of the solution for today’s global health challenges. These benefits extend beyond health delivery in low- and middle-income countries (LMICs). International volunteering, exchanges and structural placements benefit the NHS through an innovative workforce that develops international best practice and promotes lifelong learning [1]. The programmes are managed by health partnerships, defined by the Tropical Health Education Trust (THET) as “a model for improving health and health services based on ideas of co-development between actors and institutions from different countries. The partnerships are long-term but not permanent and are based on ideas of reciprocal learning and mutual benefits [2] .”

This paper discusses several models in the existing literature that could be used to measure the monetary value of the benefits from healthcare volunteering. There is an extensive literature that recognises a broad range of outcomes, mostly beneficial, which returned international volunteers bring to high income countries, including three systematic reviews [3–5]. Moreover, these outcomes have been linked to the competency frameworks designed by UK medical schools and NHS employers [3, 5]. Therefore, this literature demonstrates the link between competencies acquired in international volunteering and staff productivity as designed by the NHS appraisal process. Nonetheless, two main gaps remain to bridge these proven outcomes with the monetary value of its resulting productivity improvement. First, there is a lack of quantitative studies demonstrating the achievement of outcomes by international volunteers in the UK; some existing studies measure outcomes for international volunteers from the U.S. and Japan. Second, there is a need to define which outcomes are relevant from an NHS perspective to be included in a definition of productivity and to estimate its monetary value. For example, the type of outcomes reported in the literature extend beyond the NHS perspective, including outcomes in the domain “personal satisfaction and interest” [3]. This study aims to fill the second gap in the literature by applying a novel approach to: (1) identify from the literature the various benefits to the NHS from returned international healthcare volunteers and justify which ones can be included as key aspects from an NHS productivity perspective; and (2) quantify those benefits in monetary terms.

Benefits to the NHS from returned international healthcare volunteers are analysed within a multisector framework that encompasses all the key aspects of the economic, political, and institutional context for job creation. This analytical framework, designed by the World Bank, is known as MILES (Macroeconomic conditions, Investment climate and infrastructure, Labor market regulations and institutions, Education and skills development, and Social protection) [6]. Only three out of the five domains are relevant to capturing productivity gains from international volunteering programmes from an NHS perspective: (1) Investment climate and infrastructure, (2) Labor market regulations and institutions, and (3) Education and skills development. This study applies productivity indices to measure the effects of skills improvement on the NHS labor force.

The quantitative model of productivity indices are applied using data from health partnerships organised by King’s College London (KCL) and the Royal College of Paediatrics and Child Health (RCPCH) which placed 279 volunteers mainly in sub-Saharan Africa, including during the Ebola outbreak in 2013–2016. The model relies on the assumption that international volunteers from these partnerships are comparable with NHS staff in terms of professional occupation, and some of the volunteers develop their skills during the placement period. And additional assumption is that these volunteers return to their previous health professional category after the placement so that their learning outcomes are captured by the NHS instead of resulting in job promotion. The limitation of these assumptions is discussed.

The rest of the paper is organised as follows. Section 2 describes and maps the outcomes from international volunteers according to the MILES framework and presents the productivity valuation method used to monetise the outcomes. Section 3 explains the data and how they are applied in the quantitative model. Section 4 presents the results. The limitations of the scope of the study and from the underlying assumptions are discussed in Section 5. Finally, a discussion and concluding remarks are presented in Sections 6 and 7, respectively.

## Methods

The first step towards defining and monetising productivity gains rendered to the NHS by returned international volunteers is to understand the competencies that affect their productivity. The concept of productivity is broad but limited to the NHS perspective. This underlying productivity captures how the NHS marshals their available inputs to improve patients’ health. The main input is labor, but as well as considering the economic value of working hours, we consider outcomes that affect the organizational context and the skills of NHS staff. We discuss these outcomes within the MILES analytical framework.

### MILES framework for outcomes of international volunteering

The World Bank uses the MILES framework in designing labor market strategies by identifying key constraints for job creation within five components [6]: (M) Macroeconomics: Economic growth and net job creation, (I) Investment climate: Incentives for employers to invest and create jobs, (L) Labor market: Regulations affecting job search, hiring process and cost, (E) Education: Individual education, socioeconomics, and demographics, and (S) Social Protection: Security against loss of earnings, unemployment insurance.

In particular, MILES was used to evaluate World Bank and International Finance Corporation (IFC) programmes on youth employment [7]. We consider that the interventions studied in the World Bank/IFC portfolio share most of the characteristics with the international volunteering programmes and we therefore also apply the same domains: I, L, and E, as the domains that matter to that matter to measure impact of the programme international volunteering programmes from an NHS perspective.

We have searched literature focusing on outcomes of international volunteering in the health sector. Two recent peer-reviewed systematic reviews analyse the outcomes reported by international volunteers and how these outcomes render benefits in the UK. The study by Jones et al. [3] reviews benefits and costs of health partnerships which are classified into seven domains. Six of these domains are mapped to the key outcome indicators or competencies for five different UK professional development structures, including the NHS Knowledge & Skills Framework (KSF) and there is a recommendation to integrate volunteering within partnerships into NHS Continuing Professional Development (CPD) frameworks. The second systematic review and meta-synthesis by Tyler et al. [5] presents a more granular set of outcomes, with a core set of 133 outcomes validated by stakeholders through Delphi methods. Some examples from this core set of outcomes set are mapped to the seven dimensions by Jones et al. [3], and these can in turn be mapped to professional competency frameworks. Tyler summarised non-clinical learning outcomes under four broad themes and related to NHS policy documents and competencies [8]. The four themes are leadership, communication, cultural knowledge/skills/attitudes, and personal development. Regarding outcomes under the personal development theme, although they are related to personal satisfaction, Tyler states that these are relevant for the achievement of the NHS “6 Cs” established to ensure high quality nursing practice: care, compassion, competence, courage, commitment and communication [9].

**Fig. 1:**
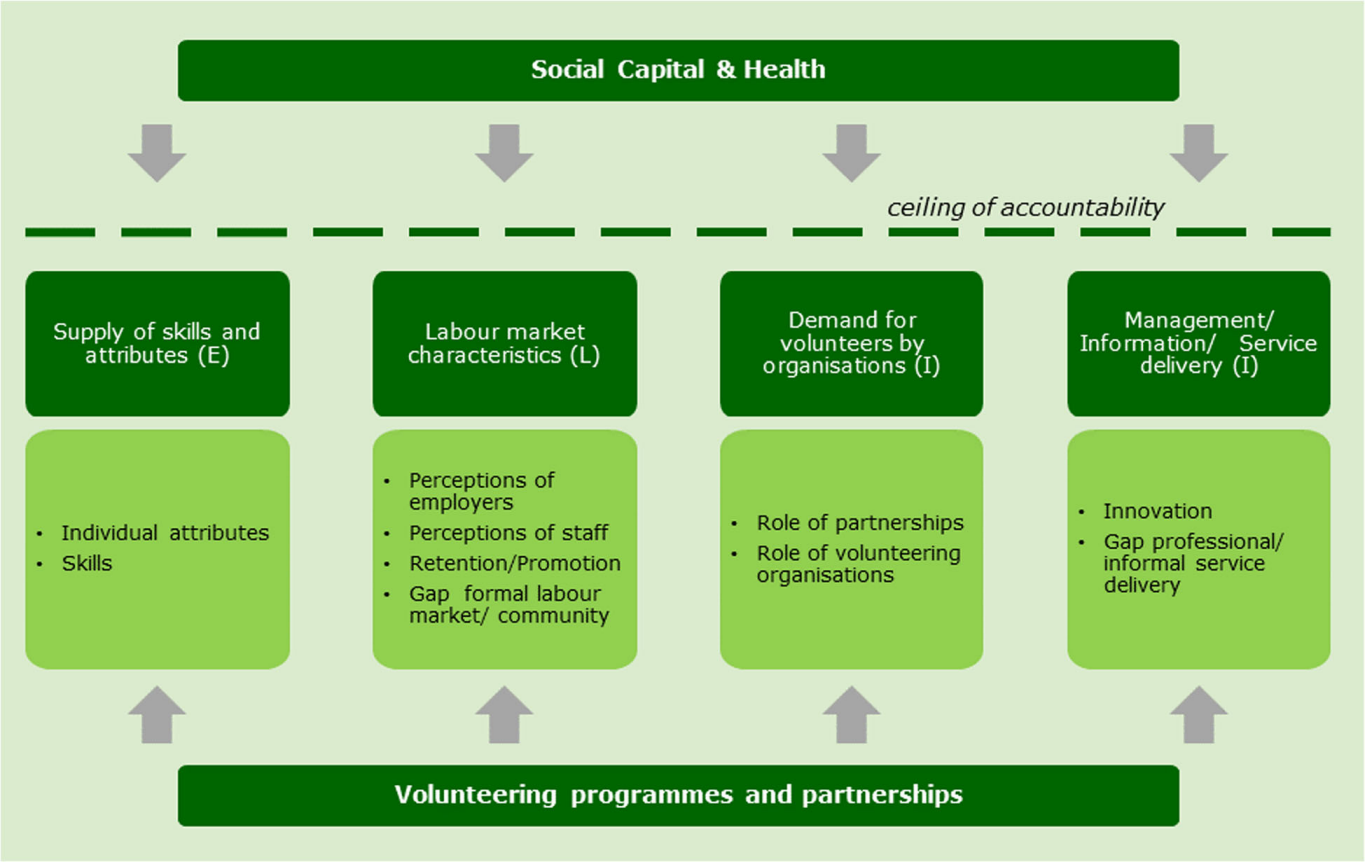
MILES OUTCOMES FRAMEWORK FOR INTERNATIONAL VOLUNTEERING

Figure 1 presents the dimensions of the adapted MILES framework. The re-classification of competencies and outcomes from [3, 5] into the I, L, E dimensions helps us to understand the link with economic value for these outcomes as generated in the process of improving productivity.

Given its link with professional competencies, all outcomes from international volunteering could be transmitted to benefits for health professionals, organisations, and ultimately to overall NHS service delivery and health outcomes for patients. This transmission of outcomes has been mapped for different domains except the domain of “personal satisfaction and interest” [3]. Nonetheless, we consider that some outcomes are more relevant to the workforce in terms of skills and job creation and should be considered under the MILES dimensions E (e.g. domain clinical skills in [3] and L (e.g. domain communication and teamwork in [3]. On the other hand, many contextual variables that affect the transferability of skills (e.g. protocols, procurement process) and that are in turn affected by outcomes such as those under “service/policy development & implementation” [3], affect job creation and productivity through their effect on the I domain.

On the I domain, outcomes are captured by healthcare organisations and help to shape relationships with donors and define the organisational role in the demand for volunteers. Recognised outcomes include improving health care service delivery in rural or difficult to reach populations, bringing healthcare management closer to the needs of local populations through decentralised management, and improving patient-provider relationships, in particular by helping to bridge the communication gap between professionals and patients. Analysed the role of international volunteering sending agencies on volunteering outcomes depending on how these agencies negotiate local dynamics and transnational governance. According to the Measuring the Outcomes for Volunteering for Education (MOVE) study, these outcomes correspond to the organisational and contextual variables facilitating or hindering the optimal acquisition and mobilisation of knowledge and core outcomes such as “appreciation of clinical governance within NHS” [5].

On the L domain, returned volunteers help improve outcomes related to career progression and attitudes of employers to apply the learning from the volunteering experience [10]. For example, it has been found that international volunteering experience favours career choices in international development and social care. UK workforce planners benefit from utilising developing country models for worker substitution, mobilisation, recruitment, and retention. In low resource settings, knowledge base on community workforce policy, training, and education are more important than those that apply in acute care and settings. Tyler et al. [5] also report “reduction in NHS drop outs” (increased staff retention) among the core outcomes.

On the E domain, the specifics of the acquired knowledge and attitudes reported by returned volunteers broadly map onto the strategic objectives of the NHS with emphasis on resourcefulness, cost efficiency, flexibility and inter-professional working [3, 5, 11–13]. Among acquired skills and attitudes, the studies cite inter-culturalism (social, cultural capital, and social entrepreneurship), improved communication, improved employee morale, enhanced problem solving, leadership skills (e.g. influencing), clinical skills applied in low resource/low technology settings, training in public health, health financing and administration, and a greater commitment to civic engagement and collaborative research. On the employer’s side, it has been reported that most UK employers perceive innovative ways such as innovation in mobile phone use, monitoring/evaluation, client tracking systems, education in communicable disease control.

The outcomes explained above and included in all studies analysed in systematic reviews [3, 5] are perceived outcomes as self-reported by international volunteers or Delphi stakeholders. To quantify performance of achievement of these outcomes, a psychometric assessment tool is being developed as part of the MOVE Project [5, 11]. To quantify performance of achievement of these outcomes, a psychometric assessment tool is being developed as part of the MOVE Project [5, 11]. To the best of our knowledge, quantitative measures of performance outcomes for international volunteers have only been presented for Japan according to a method based on “affective appraisal” or self-reported emotions [14]. Studies in the volunteering field, both local and international, have rarely examined performance outcomes of volunteers beyond satisfaction and commitment (e.g. hours of volunteering) [15].

The development and acquisition of competencies from the volunteering experience is complex and may take longer than 1 year to develop, varying by amount of individual experience and prior experience [14, 16]. The quantitative analysis of the development of competencies has assessed their impact on outcomes over time. Analysed survey data from Japanese international volunteers and predicted perceived volunteer achievement and outcomes for counterpart organizations but at three different stages of volunteering (before, during - with 1 year experience, and after). The findings point out those who reported higher levels of intercultural negotiation perceived greater achievement at the end of their volunteering service. This is also consistent with U.S. findings on the acquisition of internationally oriented outcome categories: international awareness, intercultural relations, international social capital, and international career intentions.

Figure 1 presents an umbrella domain as “social capital and health” which captures wider societal benefits derived from formal opportunity structures or activities where individual actors develop social ties and social networks, and also the value to volunteers of increased life satisfaction, achievement and personal health. International volunteer outcomes include those in the domain “personal satisfaction and interests” which pertain to “social capital and health”. However, these are not mapped onto professional competencies [3].. Although the value of these outcomes is large according to the Well-being theory [17], we apply the concept of “ceiling of accountability” as termed in the Theory of Change to present the framework from the NHS perspective in the sense that we assume that these outcomes constitute systemic factors upon which there is little control, and we focus on what can be done according to outcomes in the I, L, and E domains.

On the negative side, international volunteers have reported some risks and negative outcomes associated with the experience. Some volunteers have reported that they have “lost ground” and find their skills out-of-date, and especially the loss of networks, with some UK employers concerned about this knowledge loss or not valuing the international experience. Tyler et al. [5] find that none of the relative low number of negative outcomes has been validated by consensus from Delphi stakeholders.

### Methods of measuring monetary value of volunteering

Different theories have been put forward to measure the value of volunteering, both community and international, although these theories seek to measure value captured by the country where the volunteering activities are delivered. In particular, the International Labour Organisation’s Manual on the measurement of volunteer work recommends capturing the “economic value” of volunteering, which derives from the domestic product accounting concept applied to the “replacement theory”; basically, volunteers are considered as employment substitutes. This concept of economic value is very narrow and would only capture a part of the value of good and services supplied over the amount of volunteering time. The replacement theory overlooks two other main components of economic value: organisational benefits beyond staff wages, and quality improvements of goods and services. These components also pertain to economic value from an added value or GDP perspective and should be accounted for.

Beyond the economic value, the well-being theory and social capital theories capture wider societal benefits for the volunteers and the community. Nonetheless, none of these theories can be applied to specifically measure the value from returned volunteers from an NHS perspective. As pointed before, the outcome category under which these benefits are considered is “personal satisfaction and interest” [3] which is not mapped to recognised professional competencies.

**Fig. 2:**
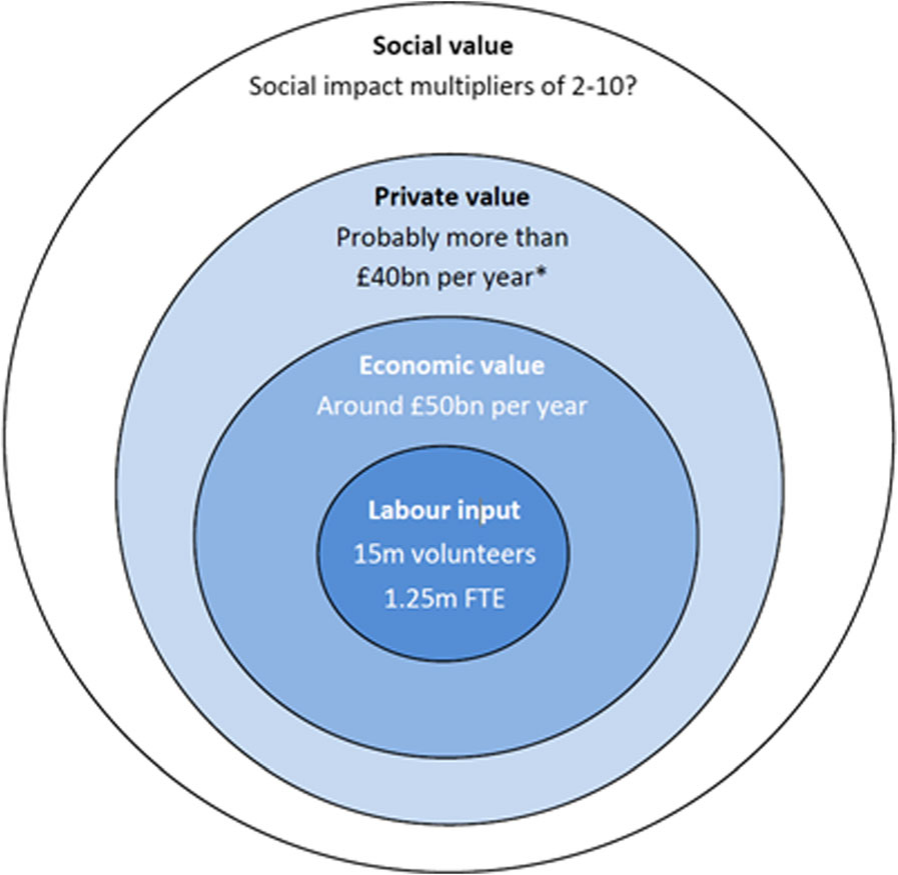
Measures of Value of volunteering: The “volunteering onion” (Source: Haldane, 2014 [18])

The “volunteering onion” as named by Haldane [18] describes several layers of volunteering value, from mere labor-substitution value (replacement theory) up to wider societal impact (social capital) (Fig. 2 and Table 1). In these terms, the outcomes in the MILES dimensions E, I and L could be considered as contributing to the value of health services produced by the NHS. The related outcomes create economic value whose measure is not straightforward since NHS services do not have market prices. Moreover, the concept and measure of economic value is indifferent to who captures it: either the workforce or the organisation, given that economic value aggregates wages and profits. Therefore, economic value, as GDP concept, incudes private returns of the international volunteering experience if this affects career progression. Yet, these private returns do not count as economic value captured by the NHS. In contrast, the two external layers of the volunteering onion: “private value” and “social value” are not captured by the economic value or measured in the country GDP. Even some theories provide a monetary valuation of improved wellbeing associated with volunteering [17], the international volunteering outcomes associated to this wellbeing or personal satisfaction cannot be mapped to current NHS professional frameworks and are beyond NHS accountability.

**Table 1:** Definition of value of volunteering in the “volunteering onion” (Fig. 2)

	Current models
Labor input	Number of volunteers
Economic Value	Replacement theory: the value of the labor input *- VIVA: The Volunteer Investment and Value Audit* [30] - ILO Manual on the measurement of volunteer work [31]
Private value	Well-being theory: the value of increased life satisfaction, including learning, achievement and personal health [17]
Social value	Social capital theory**:** benefits derived from formal opportunity structures or activities where individual actors develop social ties and social networks

Measuring productivity within the health sector is complex, including difficulties in measuring variation in skills [19] and the effects of skill-mix changes on productivity [20]. We propose a standard measure applied to labor productivity and a measure of staff quality in the NHS [19, 21]. In this sense, the economic value from the skills and attitudes brought by returned volunteers is partly captured by the NHS through improvement in staff quality/productivity.

We propose a direct measurement of NHS labor growth which has been used to quantify the impact on NHS employment in a given year. The key assumption is that the international volunteering experience causes changes in the staffing mix towards higher levels of productivity which can be captured by the earnings differentials between adjacent staff groups. Even though we have reported above how international volunteering benefits career progression and employability, we assume the productivity gain is retained by the NHS as additional economic value instead of being fully captured by returned volunteers through job promotion. Table 2 presents the links between outcomes, I, L, E dimensions, the concept of monetary value measuring associated improvements in these outcomes and dimensions, and whether this improvement is accounted for as productivity improvement from an NHS perspective in out model. Arguably, all outcome dimensions, including clinical skills, generate societal value beyond economic value which is not captured in the productivity measurement. Yet, only economic value is accounted when these outcomes and competencies are considered as part of the NHS competencies and appraisal process.

**Table 2:** Relationship between outcomes, MILES dimensions, monetary value, and productivity

Perceived outcomes		Key aspects of job creation/quality	Model of Monetary value which accounts for these outcomes (*)	Included in NHS productivity gains
Dimensions in Jones et al. (2013)	Examples of core outcomes in Tyler et al. (2018)	Dimension in Adapted MILES (Fig. 1)		
Clinical skills	- Ability to use a broader range of clinical skills - Increased awareness of/knowledge about tropical diseases - Increased awareness of/knowledge about the cultural aspects of health	E: attributes and skills	Economic value	Yes
Management skills	- Ability to be adaptable in leading - Ability to work within a system with unfamiliar power dynamics - Ability to manage projects	E: attributes and skills I: innovation	Economic value	Yes
Communication and teamwork	- Understanding that words and behaviors can have different meanings - Ability to co-operate - Ability to work as part of a team	E: attributes and skills L: perceptions/retention/gap to community	Economic value	Yes
Patient experience and dignity	- Understanding own potential to empower people - Increased respect for other cultures - Appreciation of free universal health	E: attributes and skills L: perceptions/ gap to community I: role NHS and partnerships/ gap professional-informal service delivery	Economic value	Yes
Service/policy development and implementation	- Increased awareness of/knowledge about the positive impact of clinical policies and governance - Appreciation of excellent human resource in the NHS	I: role of NHS and partnerships	Economic value	Yes
Academic skills	- Ability to dissemination best practice globally - Improvement in teaching skills - Ability to build a global network	E: attributes and skills, L: perceptions/ gap community I: role NHS and partnerships	Economic value	Yes
Personal satisfaction and interests	- Ability to develop friendships - Refreshment and reinvigoration - Can-do attitude	Social capital and health	Private value (Well-being theory) Social value (Social capital)	No

To measure labor growth attributed to international volunteers we use the proposed method of an index of input growth which weights the number of staff of each type by their respective wages. We use baseline constant wages in 2017 reported in the NHS Staff Earnings statistics as weights for both the pre- and post-volunteering periods. By holding wages constant, our index of input growth is a Laspeyres index which measures “real” changes in the weighted volume of NHS staff caused by the international volunteering experience; we do not measure how much these volunteers are paid. Nonetheless, this real or quality-adjusted growth in volume of NHS staff can be monetised according to average wages to provide a monetary value of the benefits for the NHS from returned international volunteers.

The Laspeyres volume index, in which inputs are measured directly, takes the following form:

1 $$ \Delta  Z=\frac{\sum \limits_{n=1}^N{z}_{nr}{w}_{nb}}{\sum \limits_{n=1}^N{z}_{nv}{w}_{nb}} $$

Where *z*_*nv*_ denotes the number of international volunteers whose NHS staff group is n, and *z*_*nr*_ denotes the number of returned international volunteers as ascribed to a productivity level of staff group n. Wages for each staff group n, *w*_*nb*_, are measured for the annual period b (fiscal year 2016/17). The “real” or “quality adjusted” changes applied to NHS staff from their international volunteering experience is (*∆Z* − 1)% in terms of increase in quality-adjusted staff volume. This percentage increase can be measured separately for different staff groups and a monetary value can be calculated in proportion to the average earnings of the staff group. The monetary benefit could be measured as the product of the baseline value by the productivity growth index: $\left(\sum \limits_{n=1}^N{z}_{nv}{w}_{nb}\right)\left(\Delta  Z-1\right)$.

We do not have an exact definition of FTE volume of volunteers of type n (*z*_*nv*_), so some sensitivity analysis is carried out from alternative definitions, in particular to the adjustment of FTE from observed volunteer days so that the productivity gain of a given volunteer is increasing with the volunteering time, which corresponds to findings of increased achievement outcomes as predicted by competencies acquired during the volunteering experience [14].

The productivity gain corresponding to each of the three scenarios when converting volunteering days (D) to annual FTE time, are calculated as follows:

### Scenario 1: productivity gain proportional to volunteering time.

This scenario assumes that the gains accrued from international volunteering are proportional to the volunteering time and no larger than annual productivity premium in cases of volunteering periods longer than 1 year. Therefore, the index of annual productivity growth (1) is calculated as:

2 $$ \Delta  Z=\left\{\begin{array}{c}\frac{\sum \limits_{n=1}^N\left(\frac{D}{260}\right){z}_{nr}{w}_{nb}+\left(1-\frac{D}{260}\right){z}_{\mathrm{n}v}{w}_{nb}}{\sum \limits_{n=1}^N{z}_{nv}{w}_{nb}}, if\ D\le 260\\ {}\frac{\sum \limits_{n=1}^N{z}_{nr}{w}_{nb}}{\sum \limits_{n=1}^N{z}_{nv}{w}_{nb}}\  if\ D>260\end{array}\right. $$

This scenario becomes a pessimistic or conservative assumption when valuing productivity gains for short-term volunteering experiences.

### Scenario 2: annual productivity gain from all volunteering experiences.

This scenario assumes that all volunteers achieve full productivity gains independently of their volunteering time. In this sense it is an optimistic scenario for short-term volunteering experiences.

In this case, the index is applied in its simple expression (1) considering all volunteers as FTE returned volunteers accruing FTE productivity gains.

### Scenario 3: annual productivity gain from volunteering experiences longer than 1 month.

There are arguments on how the length of volunteering time affects the returns for returned volunteers both positively and negatively [11]. Given our dataset with many short stay volunteers and fewer long term volunteers, we adjust the index of productivity growth to consider a lower threshold of 1 month (22 volunteering days) to benefit from the productivity gains of the volunteering experience. In this case, the productivity growth index is calculated as

3 $$ \Delta  Z=\frac{\sum \limits_{n=1}^N\overline{z_{nr}}{w}_{nb}+\sum \limits_{n=1}^N\underset{\_}{z_{nv}}{w}_{nb}}{\sum \limits_{n=1}^N{z}_{nv}{w}_{nb}} $$

All productivity gains are attributed to volunteers in the input $\overline{z_{nr}}$ which corresponds to volunteers with experiences of 22 days or more whose productivity gains are accrued from the assumed transition of productivity from staff group *z*_*nv*_ to *z*_*nr*_. Volunteers who stay less than 22 days are inputted as $\underset{\_}{z_{nv}}$, that is, at the same staff group and salary as in the pre-volunteering experience.

This scenario functions as an intermediate assumption, even though it is more conservative than the first scenario for very short stays which are more common among senior staff.

## Data

Three health partnerships from King’s College London (KCL) and one from the Royal College of Paediatrics and Child Health (RCPCH) have provided data of volunteers. The KCL partnerships placed volunteers in Sierra Leone (King’s Sierra Leone Partnership - KSLP), Somalia (King’s Somaliland Partnership – KSP), and Democratic Republic of Congo (King’s Congo Central Partnership – KKCP). RCPCH operates in a wide range of LMICs; the sample of volunteers were placed in six countries (Cambodia, Ghana, Kenya, Myanmar, Sierra Leone, and Uganda). Details of the activities of these partnerships are provided in Additional file 1.

Table 3 below can give the reader an idea of the volunteers involved in these types of initiatives. We have gathered data from a total of 279 volunteers who have volunteered overseas, and included 272 volunteers in the database, excluding 5 volunteers whose role was not specified, and 2 retired volunteers.

**Table 3:** Number of volunteers and average (mean) volunteering time in days

	All partnerships		KKCP		KCSLP		KSP		RCPCH	
	Number	Average time (D)	Number	Average time (D)	Number	Average time (D)	Number	Average time (D)	Number	Average time (D)
HCHS Doctor	185 (68%)	281.6	3	12.3	65	170.2	19	9.6	98	416.4
Nurses	45 (17%)	128.4	3	12.7	26	199.6	14	13.6	2	180.0
Others	42 (15%)	227.2	1	15.0	37	259.1	4	7.3	0	
Total	272 (100%)	247.8	7	12.9	128	201.2	37	10.9	100	411.7

(*) Volunteering time imputed for 30 volunteers with missing information in KCSLP: 11 HSHC doctors, 4 Nurses, and 15 Other

King’s College programmes: KKCP, KCSLP, KSP

Royal College of Paediatricians programme: RCPCH

Our dataset of international volunteers from KCP and RCPCH includes information on the number of volunteers, volunteering function, and volunteering time in days, which are the variables needed to compute the productivity indexes. The allocation of a NHS staff group, occupation and care setting requires some ad-hoc assumptions to match the information in the dataset with the definitions of staff group, occupation code, level and care setting available from NHS Staff Earnings statistics. For example, in the KCSLP the volunteer function describes the volunteer role in the host country (e.g. Ebola nurse, Nurse educator, doctor) and we make several assumptions to match these roles with the NHS staff groups and occupations. Therefore, although the matching of these levels has considered the role definitions and HEE core skills and competencies, some margin of arbitrariness remains. On the contrary, the KKCP database exactly specifies the volunteering role as a defined NHS staff group (e.g. Specialty Registrar), which we adopt as the pre-volunteering category.

On the volunteering time, some missing information in the KCSLP programme, has been replaced with average days in the corresponding category from non-missing data, with three considered categories: Hospital and Community Health Services (HCHS) doctors, Nurses, and Others. In total, volunteering time has been imputed for 30 volunteers with missing information from a total of 128 volunteers in this programme. We tested the sensitivity of our results to this imputation by undertaking the calculations without including these 30 missing observations.

A top-level comparison of our sample of volunteers to that from the MOVE study [11] in terms of staff categories and volunteering time presents a similar distribution. The most numerous group also corresponds to doctors (HCHS), followed by nurses and midwives. Doctors are more represented in our sample (68%) than in MOVE (36%) due to the large participation of paediatricians in the RCPCH partnership. The representation of nurses and midwifes is similar (17% of our sample versus 21% of MOVE sample). Therefore, there are relatively less volunteers under our ‘Others’ group but as well as in MOVE most of them are classified as support to clinical staff and Allied healthcare professionals (e.g. therapists).

Regarding volunteering time, just a few of our sample volunteers (from KSP) can be considered as short term (under 1week) volunteers according to MOVE definition. The average days of volunteering in our sample is 248.7 days, and this average is above 3 months in our three staff categories, corresponding to a larger representation of long-term volunteering than in MOVE. This is due to long-term placements by KCSLP and RCPCH volunteers.

Annual wages data for different staff groups, occupations, levels, and care settings have been collected from NHS Digital, Provisional NHS Staff Earnings Estimates. Wages presented for the annual period October 2016 to September 2017 [22, 23].

## Results

The estimations of productivity indexes (1), (2), and (3) are presented separately for the four group of volunteers and for three broad staff groups: HCHS doctors, Nurses, and Other. Comparisons across groups are presented in terms of the percentage point increase in “real” volume of NHS staff and of average monetary value of productivity gain per volunteer per year. These comparisons will shed light on the effect of different scenarios depending on the observed volunteering time and staff mix allowing an assessment of which is the most plausible scenario and results.

Table 3 presents the average time of volunteers in each of the four programmes. The volunteers from the RCPCH programme, 98% in the HCHS doctors staff group, have volunteering periods longer than 1 year. The King’s College programmes vary in terms of average volunteering time, with average volunteering periods close to 1 year for the Sierra Leone partnership and around a fortnight for the Somaliland and DRC programmes.

With regard to the monetisation of volunteering benefits, Tables 4 and 5 summarise the productivity growth index and the consequent monetary benefits per volunteer. Scenario assumptions are only relevant for KKCP and KSP whose volunteers have average stays below 2 weeks. In contrast, the RCPCH programme benefits do not change significantly when comparing the conservative scenario 1 and the optimistic scenario 2, which coincides with scenario 3 since the 1 month threshold is met by all volunteers. According to the MOVE study, 84% of volunteers have a length of placement over 2 weeks so that the optimistic and conservative scenarios should be more realistic to measure productivity.

**Table 4:** Productivity growth index

	KKCP			KCSLP			KSP			RCPCH	
	Scenario			Scenario			Scenario			Scenario	
	1	2	3	1	2	3	1	2	3	1	2 and 3
HCHS Doctors	0.7%	19.2%	2.2%	19.4%	37.2%	35.7%	1.0%	26.4%	0.0%	31.9%	41.0%
Nurses	3.0%	62.1%	0.0%	33.0%	56.1%	53.0%	1.0%	22.0%	2.2%	39.8%	57.4%
Others	5.4%	93.9%	0.0%	26.9%	30.2%	30.2%	0.3%	12.1%	0.0%	n.a	n.a
Weighted average	1.8%	38.9%	1.3%	22.8%	38.2%	36.8%	0.9%	24.3%	0.5%	31.9%	41.2%

Scenario 1 (Conservative): productivity gain proportional to volunteering time

Scenario 2 (Optimistic): Full productivity gain for all volunteers

Scenario 3 (Intermediate): Full productivity gains for volunteers with one month or more volunteering experience

**Table 5:** Monetary value of productivity increase per volunteer

	KKCP			KCSLP			KSP			RCPCH	
	Scenario			Scenario			Scenario			Scenario	
	1	2	3	1	2	3	1	2	3	1	2 and 3
HCHS Doctors	£386	£11,326	£1313	£11,329	£21,780	£20,884	£723	£19,140	£0	£20,261	£26,099
Nurses	£927	£19,034	£0	£9893	£16,835	£15,882	£353	£7798	£777	£12,352	£17,841
Others	£1325	£22,963	£0	£9122	£10,245	£10,245	£97	£4027	£0	n.a	n.a
Weighted average	£752	£16,292	£563	£10,399	£17,441	£16,793	£515	£13,215	£294	£20,102	£25,934

Scenario 1 (Conservative): productivity gain proportional to volunteering time

Scenario 2 (Optimistic): Full productivity gain for all volunteers

Scenario 3 (Intermediate): Full productivity gains for volunteers with one month or more volunteering experience

The RCPCH programme is the most beneficial for HCHS doctors whose increase in productivity is 41%, valued at £26,099. The explanation of this advantage with respect to other programmes is due to the case-mix of HCHS doctors which in the RCPCH programme is assumed to be homogeneous for Specialty Doctors (mean annual earnings of £63,586) whose productivity gains are imputed by new skills equivalent to the upper Associate Specialist productivity level (mean annual earnings of £89,685). For other programmes, the average productivity gain of HCHS doctors in the optimistic scenario 2 ranges between 19 and 37%, with average benefits between £11,326 and £21,780, according to a staff mix which includes doctors in levels below those of Specialty doctors.

Volunteer nurses have larger benefits than doctors except in the KSP programme. This benefit implies an annual productivity increase of up to around 60%, valued at £19,034, for the KKCP partnership in scenario 2. The differences in productivity gain are due to the nurse occupational category and levels. At the lower level, the KSP programme includes several nurses in the educational care setting, and some 2nd level nurses for which adjacent levels to 1st level nurses or even to nurse consultant implies annual salary increases below £3000. At the upper level, all nurses participating in the KKCP program are ascribed to the Acute, Elderly & General care setting and their volunteering experience is assumed to benefit initial 1st level nurses with the skills of nurse managers. The development of these skills is valued at £19,034 per nurse according to the difference in annual earnings between nurse managers and 1st level nurses.

The only programme which represents volunteers in clinical staff and NHS infrastructure support roles is the KCSLP programme which includes 37 volunteers in this group, mainly in scientific and technical positions. These volunteers accrue around 30% in productivity gains to the NHS, which represents £10,245 per year.

These results are very slightly affected by the imputation of volunteering time for missing data in the KCSLP. The maximum absolute effect of this imputation of time is an overestimation of 5% in the value per volunteer in scenario 1, such that is missing data are not replaced with average time per category, the overall value at £10,399 per volunteer would decrease to £9862.

Overall, we could conclude that if the acquisition of volunteering outcomes is realised, the NHS can accrue between 24 and 41% of productivity increase per volunteer with a value between £13,215 and £25,934 per volunteer.

## Limitations

Limitations of this study refer to the identification of achievement outcomes and consequent monetary measure as particular from international volunteering programmes. That is, to what extent is the achievement of these outcomes, and the associated monetary value of the productivity improvement different from other training programmes, e.g. domestic programmes in the UK?

Regarding the achievement, we have cited some longitudinal and panel data studies from the U.S. [16] and Japan [14] proving the achievement of different competencies which are particularly and better acquired during the international volunteering experiences. Although Onuki points as limitation to measure causal impact the lack of a comparison group, we think that the assessment of learning outcomes over time goes beyond mere association and further psychometric work as expected from the MOVE study will confirm such impact.

The monetary value of the productivity improvement we have presented does not aim to be especially designed for international volunteers. Yet, it follows the logic of measuring quality improvement for labor applied to a minimum skill improvement as defined by promotion to adjacent categories. These productivity gains are captured by the NHS through improved services at no extra cost in terms of salaries. We do not rule out a positive effect of international volunteering on career progression, but this is not a frequently reported outcome. Any ad hoc assumption on career progression would not change the quantitative results. Yet, the interpretation of productivity gain should be monetised as a private return for international volunteers instead of a benefit for the NHS.

An additional limitation is the blurred boundary between the societal and the NHS perspectives. We have argued on a ceiling on accountability so that the NHS is not accountable for competencies related to personal satisfaction and interest. Nonetheless, we have also mentioned that these personal traits are also related to desirable competencies (the 6Cs). Moreover, each of the outcomes can have societal spillovers over and above economic value and for non-health sectors, as it happens with any outcome contributing to health gains.

## Discussion

### Implications of study results

The study results suggest that international volunteering generates average productivity gains of up to 37% for doctors and un to 62% for nurses. Average productivity gains estimated from health partnerships data vary depending on the duration of the volunteering period as well as in the occupational category mix. These gains are monetised based on 2017 NHS staff earnings data.

Overall, we conclude that if the acquisition of volunteering outcomes is realised, the NHS can accrue a productivity increase of between 24 and 41% per volunteer, with a monetary value ranging from £13,215 to £25,934 per volunteer.

### International and UK context of the global engagement strategy

To understand the role of health partnerships and its benefits, it is important to understand the Global Engagement strategy of HEE (HEE: forthcoming) within the global health strategy and the role of official development assistance (ODA) and into the UK Aid strategy. This global health strategy is also linked to the current trends in NHS competencies and the importance of international volunteering outcomes within them.

The *Lancet* Commission on Investing in Health [24, 25] demonstrated the importance and reflected on the future of ODA for health and its role in achieving grand convergence and the health-related Sustainable Development Goals (SDGs). The introduction of sustainable development changed the paradigm of poverty reduction, the overriding objective of the former Millennium Development Goals (MDGs); “Sustainable development is about all of us, not some of us. It is about taking the health of future generations as seriously as we take our own. And it is about rethinking the economic models on which our present highly consumptive societies depend. The kind of economy one needs to deliver sustainable and inclusive development is likely to be very different from the economy of today.” [26].

Health is only entirely targeted in one (third SDG) out of 17 SDGs. However, this does not mean that health has been deprioritised in the development agenda considering that three of the MDGs were dedicated to health. All SDGs are connected, and most can be linked back to health [27]. In particular, international volunteering and placements organised by health partnerships can be considered as a target of SDG 17 (partnerships for the goals) in terms of accountable and well-functioning institutions with multiple stakeholders from developed (sender) and developing (host) countries, with a stewardship role for the WHO on global health.

The implementation of Universal Health Coverage (UCH) as a target of the SDG 3 has been organised around international health partnerships. The fundaments of the partnership between institutions of LMICs are defined by the health strategy put forward by LMICs, and donors remain accountable on its achievements. Therefore, LMICs are empowered in defining their health strategies and accountability by health partnerships removes the element of discretion which can result from earmarking donor funds to specific programmes. International Health Partnerships for UHC 2030 has evaluated performance of partnerships in this sense [28]. One of the recommendations of the most important organisation managing health partnerships in the UK, THET, to the UK government states “Supporting future health partnership programmes to explore models of increased ownership by LMIC partners whilst still ensuring good value for money and quality grant management. This has many potential benefits and could help address structural barriers to securing mutual benefit such as transparency.” [2].

### The future of professional competencies for NHS staff

By 2013, Jones et al. [3] mapped professional competencies developed by HEE, NHS employers, and medical professional bodies. Only 5 years have passed but these professional competencies have been updated according to the underlying themes of international volunteering outcomes, that is, towards multi-disciplinarily and internationalisations (towards “global workforce” strategy outcomes). Some of the new professional competency frameworks include optional dimensions in the KSF such as develop ‘team’ objectives for large integrated teams, and a new optional leadership and management dimension for senior roles which has been developed in response to feedback from employers. The Academy of Royal Colleges has introduced the Broad Based Training programme designed to give trainees a broad experience across four specialties. The General Medical Council has developed the Generic professional capabilities framework with the Academy of Medical Royal Colleges to describe the fundamental, career-long, generic capabilities required of every doctor. The RCPCH has proposed global child health competencies for UK general/ paediatricians [29].

## Conclusions

We have discussed some studies which report outcomes perceived by international volunteers as these benefit the sender developed country patients and organisations. All these studies capture different qualitative dimensions for outcomes reported by returned international volunteers. These dimensions match learning outcomes pursued in the capacity building programmes of HEE, whose overarching aim is the development of a workforce with the skills, values and behaviors to respond to future needs and drive innovation and improvement. Evidence on the development of these skills will be collected and monitored through a psychometric tool developed by HEE and this evidence can be used to measure quality improvements in staff and organisations.

In this paper we have presented a rationale to place the global learning outcomes from international volunteering into a framework which emphasises changes in labor market outcomes for both the individual (e.g. skills and attributes) and the organisations (e.g. staff retention, innovation). We present a method to monetise these outcomes based on measures of quality-adjusted changes in NHS staff. In contrast to the method applied to real changes in staff configuration, we apply a hypothetical scenario analysis which assumes the same staff configuration as in the pre-volunteering period but a quality improvement for returned international volunteers whose monetary value is the productivity gain implicit in a superior salary level within each staff category (doctors, nurses, and other support staff).

In summary, the analysis presented here offers a valuation method only for one of the aims of international volunteering programmes: the development of the existing and future NHS workforce. Broader benefits for health system strengthening at global level are recognised but not accounted for such that our analysis offers a value for money rationale for international volunteering programmes purely from a domestic and NHS perspective.

Under the current financial pressures and shortages of healthcare staff, it is important to assess the opportunity costs of international volunteering and placements realized by NHS staff and by the potential healthcare labor force. Our analysis offers this assessment providing a value for money rationale for international volunteering programmes from a domestic and NHS perspective. The valuation method considers only one of the aims of international volunteering programmes: the development of the existing and future NHS workforce. Broader benefits for health system strengthening at a global level are acknowledged but not accounted for.

Overall, we conclude that if the acquisition of volunteering outcomes is realised, the NHS can accrue a productivity increase of between 24 and 41% per volunteer, with a value ranging from £13,215 to £25,934 per volunteer.

## Additional file

Additional file 1: Background on health partnerships [32–34]. (DOCX 34 kb)
