# Supplementary material for: The value of international volunteers experience to the NHS
Source: Global Health. 2019 Apr 23;15:31. doi: 10.1186/s12992-019-0473-y (PMC6480499; doi:10.1186/s12992-019-0473-y)
Supplement: Supplementary file 1 — Background on health partnerships [32–34]. (DOCX 34 kb) [file 12992_2019_473_MOESM1_ESM.docx]

Additional file 1: BACKGROUND ON HEALTH PARTNERSHIPS

This section gives an overview of some of the volunteering programs available, as well as a description of volunteers’ profiles and, from an NHS perspective.

Health partnerships between UK health institutions and their counterparts in other countries are usually driven by the needs of the host country, and the list of tasks and functions to be done by volunteers is usually developed in conjunction between the two parties. The nature of the deployment, the roles in the host country and the objectives and quality of the placement organisation all vary enormously.

Individuals involved in these volunteering programs are not homogenous. Both experienced experts and young professionals and students seeking to spend a period of time abroad can be seen among the different settlements.

According to general roles’ description, volunteers should have good interpersonal and intercultural skills; be eager to work within a multicultural team and with senior staff from partner institutions; know how to work independently with minimal supervision; and have an appreciation of the philosophy behind a long-term co-development health partnership.

The various international volunteering programmes define staff responsibilities and key areas of development. In particular, a description of health partnerships from King’s College London (KCL) and the Royal College of Paediatrics and Child Health (RCPCH) [32-34] is presented below, which can shed some light on the types of activities undertaken by volunteers.

KCL sees the partnerships as a model for improving health and health services based on ideas of co-development between actors and institutions from the UK and several countries in sub-Saharan Africa. The partnerships are long-term but not permanent, and are based on ideas of reciprocal learning and mutual benefits. The available programs focus on capacity building – the development of sustainable skills, organisational structures, resources and commitment to improving health. There are three ongoing partnerships:

*King’s Sierra Leone Partnership (KSLP)* [32-33]

This is a long-term health partnership between King’s Health Partners, and the College of Medicine and Allied Health Sciences (COMAHS), Connaught Hospital and the Ministry of Health and Sanitation.

During the Ebola outbreak in 2013-2016 there was a marked reduction in healthcare utilization, and significant mortality and emigration among healthcare professionals. In recent years, as Sierra Leone rebuilds its health system, the focus has moved to strengthening the health system in general with a particular emphasis on developing tertiary care and enabling a suitable referral environment both for district hospitals to escalate complex adult medical and surgical cases and for quality post-graduate specialisation to begin.

Along with other NGOs, KSLP has been working with the National HIV/AIDS Control Programme to support implementation of a new “Catch Up Plan”. King’s volunteers also work closely with NGO partners including CHAI, Welbodi Partnership and Partners in Health, and the World Health Organization Country Office.

An in-country KSLP team of between 25 and 30 people (at any one time), works across policy, service delivery, research, education and training, supported by wider academic and NHS clinical communities. Activities include but are not limited to: updating guidelines, supporting national training sessions, monitoring and supervising staff, working with hospital management to develop and implement new policies, supporting care for inpatients through clinical work with junior doctors, and collaborating with local infectious disease specialists.

*King’s Somaliland Partnership (KSP)* [32]

KSP focuses on health education and training, policy and regulation, hospital management and research. KSP has grown from a handful of volunteers, working with Somaliland’s only health training institution at the time, to an organisation with an active base of over 150 individuals working across 11 speciality areas, from paediatrics to public health. Volunteers are practising UK experts – NHS clinicians, nurses, midwives, educationalists, academics, researchers, regulators, managers and directors – who donate their evenings, weekends, holidays and sabbaticals to the Partnership. Today, the Somaliland Partnership works with 13 key Somaliland institutions and reaches hundreds of Somaliland health professionals and students annually.

*King’s Kongo Central Partnership (KKCP)* [32]

This is a long-term partnership between the King’s Centre for Global Health, the Kongo Central Ministry of Health, Hospital General de Kinkanda in Matadi and the Universite Joseph Kasa Vubu in Boma, in the Democratic Republic of Congo. KKCP aims to increase the knowledge and skills of individual health workers and implement system change to meet the growing needs of the Kongo Central population, specifically in trauma care. Current work is focused on the development of a trauma registry and running primary trauma care courses. The long-term goal of the partnership is to improve health outcomes through trauma system development, clinical care, research and training.

*The Royal College of Paediatrics and Child Health Partnerships (RCPCH)* [34]

RCPCH engages in a range of different global programmes help improve child health worldwide, working with experienced paediatricians and other child health professionals, as well as local partner organisations.

RCPCH Global has grown significantly in the last five years. They have expanded their scale of work, and consolidated the programmes strategy to focus on improving quality of clinical care for children in hospitals and health centres.

Team members work with local partners, ministries of health and international development agencies in a wide range of low- and middle-income settings, including Kenya, Uganda, Rwanda, Sierra Leone, Ghana, Malawi, Nigeria, Egypt, Jordan, Palestine, Myanmar, India, Sri Lanka and Cambodia. The organisation recruits and prepares doctors, nurses and midwives who help in all aspects of programme delivery, usually through longer-term placements overseas in the Global Links programme.

Placements include: training and mentoring of local healthcare staff, transferring skills in paediatric and neonatal care, building the capacity of the psychiatric intensive care Unit, neonatal intensive care unit, outpatient department and paediatric emergency services, conducting clinical audits and quality improvement projects.
